# 99. Impact of Covid-19, Divergent Genomic Modes of Transmission and Strain Replacement of Carbapenem-Resistant Organisms in a Single Hospital in Singapore over 12 years

**DOI:** 10.1093/ofid/ofae631.036

**Published:** 2025-01-29

**Authors:** Ze Qin Lim, Dennis Loy, Sai Rama sridatta Prakki, Natascha May Thevasagayam, Vanessa Koh, Jie Yin Chua, Pei Yun Hon, Nurhidayah Binte Mohamed Yazid, Jin Ting Ong, Xiaowei Huan, Zaw Linn Kyaw, Pooja Rao, Jeanette Teo, Indumathi Venkatachalam, Partha P De, Shawn Vasoo, Kalisvar Marimuthu, Oon Tek Ng

**Affiliations:** NCID, Singapore, Not Applicable, Singapore; NCID, Singapore, Not Applicable, Singapore; Helmholtz Munich, Munich, Bayern, Germany; NCID, Singapore, Not Applicable, Singapore; NCID, Singapore, Not Applicable, Singapore; NCID, Singapore, Not Applicable, Singapore; NCID, Singapore, Not Applicable, Singapore; NCID, Singapore, Not Applicable, Singapore; Singapore General Hospital, Singapore, Not Applicable, Singapore; NCID, Singapore, Not Applicable, Singapore; NCID, Singapore, Not Applicable, Singapore; Tan Tock Seng Hospital, Singapore, Not Applicable, Singapore; National University Hospital, Singapore, Not Applicable, Singapore; Singapore General Hospital, Singapore, Not Applicable, Singapore; Tan Tock Seng Hospital, Singapore, Not Applicable, Singapore; National Centre for Infectious Diseases and Tan Tock Seng Hospital, Dept of Infectious Diseases, Singapore, Singapore; NCID, Singapore, Not Applicable, Singapore; National Centre for Infectious Diseases, Singapore, Singapore

## Abstract

**Background:**

Much remains unknown about the different genomic characteristics of transmission comparing Carbapenem-Resistant Enterobacterales (CREs), Carbapenem-Resistant *A. baumannii* (CRAB) and Carbapenem-Resistant *P. aeruginosa* (CRPA). In this study, we examine the genomic characteristics and transmission dynamics of Carbapenemase-producing Organisms (CPOs) isolated in a single hospital from 2010 to 2022.

**Table 1.**
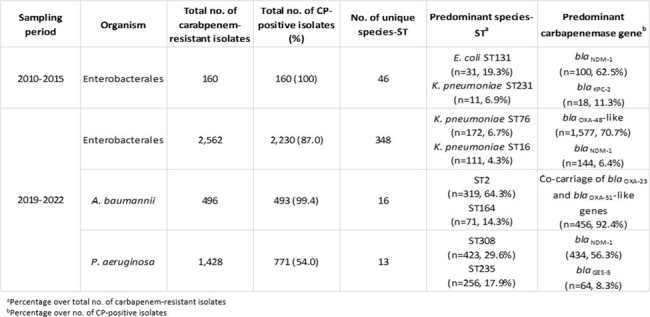
Genomic characterization of CPOs detected between 2010 and 2022

**Methods:**

All Carbapenemase-Producing Enterobacterales (CPE) (n=160) identified between 2010 and 2015 and all CRE (n=2,573), carbapenem-resistant *Acinetobacter spp*. (n=500) and carbapenem-resistant *Pseudomonas spp*. (n=1,434) identified between 2019 and 2022 were whole-genome-sequenced. An outbreak was defined as monthly count greater than 2 SD above the mean. Two or more isolates of a unique species-ST combination formed an ST-cluster.

**Table 2.**
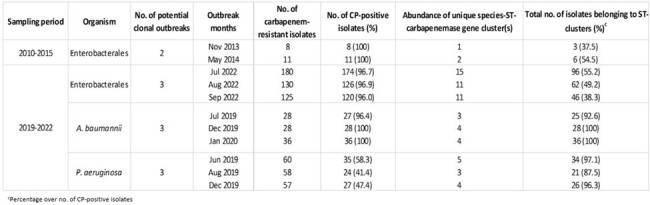
Outbreak periods among three major groups of CPOs between 2010 and 2022.

**Results:**

Of the total 4,667 WGS isolates, 2,828 (60.6%) were surveillance and 1,839 (39.4%) were clinical cultures. The predominant species-ST and carbapenemase gene detected between 2010 and 2015 was *E. coli* ST131 (n=31, 19.3%) and *bla*_NDM-1_ (n=100, 62.5%), respectively, while *K. pneumoniae* ST76 (n=172, 6.7%) and *bla*_OXA-48_-like (n=1,577, 70.7%) was the predominant species-ST and carbapenemase gene detected among CPEs isolated between 2019 and 2022 (Table 1). The majority of CRE (87.0%), CRAB (99.4%) and CRPA (54.0%) were carbapenemase-producers. During outbreaks, ST-clusters accounted for the majority of *A. baumannii* (92.6% – 100%) and *P. aeruginosa* (87.5% – 97.1%). ST-clusters accounted for approximately half of CPE outbreaks (37.5% – 55.2%) (Table 2). CRAB and CRPA numbers declined during Covid-19 (2020 to 2021) and remained low (2022) while CRE numbers increased post-Covid-19 (2022) (Figure 1).

**Figure 1.**
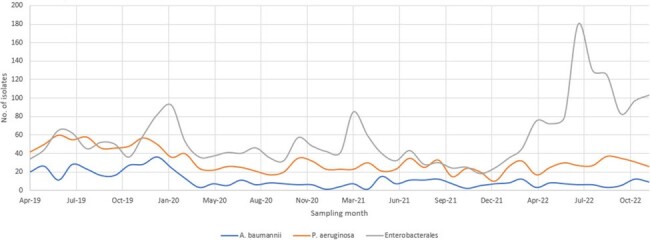
Monthly incidence rates of carbapenem-resistant Enterobacterales, A. baumannii and P. aeruginosa from 2019 to 2022.

**Conclusion:**

Clonal transmission accounted for the vast majority of CRAB and CRPA transmission while horizontal gene transfer, likely via plasmid-mediated transmission, accounted for significant CPE transmission. The varying genomic transmission modes is a possible factor for the different trends observed for CRAB, CRPA and CRE transmission pre- and post-Covid-19. Bacterial strain replacement accounted for the carbapenemase gene replacement observed over the 12-year period, possibly due to plasmid-bacterial host factors.

**Disclosures:**

**Shawn Vasoo, MBBS, MRCP, D(ABP), D(ABIM) (Inf Dis), FRCPath**, bioMerieux: In-kind, for this study|Rosco Diagnostica: In-kind, for this study

